# Prediction of Alternative Drug-Induced Liver Injury Classifications Using Molecular Descriptors, Gene Expression Perturbation, and Toxicology Reports

**DOI:** 10.3389/fgene.2021.661075

**Published:** 2021-07-01

**Authors:** Wojciech Lesiński, Krzysztof Mnich, Witold R. Rudnicki

**Affiliations:** ^1^Institute of Computer Science, University of Bialystok, Białystok, Poland; ^2^Computational Center, University of Bialystok, Białystok, Poland

**Keywords:** machine learning, random forest, data integration, drug induced liver injury, feature selection

## Abstract

**Motivation:** Drug-induced liver injury (DILI) is one of the primary problems in drug development. Early prediction of DILI, based on the chemical properties of substances and experiments performed on cell lines, would bring a significant reduction in the cost of clinical trials and faster development of drugs. The current study aims to build predictive models of risk of DILI for chemical compounds using multiple sources of information.

**Methods:** Using several supervised machine learning algorithms, we built predictive models for several alternative splits of compounds between DILI and non-DILI classes. To this end, we used chemical properties of the given compounds, their effects on gene expression levels in six human cell lines treated with them, as well as their toxicological profiles. First, we identified the most informative variables in all data sets. Then, these variables were used to build machine learning models. Finally, composite models were built with the Super Learner approach. All modeling was performed using multiple repeats of cross-validation for unbiased and precise estimates of performance.

**Results:** With one exception, gene expression profiles of human cell lines were non-informative and resulted in random models. Toxicological reports were not useful for prediction of DILI. The best results were obtained for models discerning between harmless compounds and those for which any level of DILI was observed (AUC = 0.75). These models were built with Random Forest algorithm that used molecular descriptors.

## 1. Introduction

Drug-induced liver injury (DILI) is common problem in drug development since nearly all classes of medications can cause liver disease (David, 2010; Raschi and De Ponti, 2017). An estimated 1,000 drugs have been implicated in causing liver disease (Kaplowitz, 2005). Some drugs can injure the liver, and in extreme cases therapy can be more dangerous than the disease for which they are prescribed. DILI accounts for approximately half of the cases of acute liver failure (Li et al., 2020). DILI has diverse symptoms—it mimics all forms of acute and chronic liver disease (Thakkar et al., 2019). With the exception of rare cases, DILI subsides after cessation of treatment with the drug. Nevertheless, it represents an important diagnostic and therapeutic challenge for physicians (Kaplowitz, 2004).

Multiple approaches were examined for DILI prediction. Vorrink et al. (2018) proposed an experimental approach, using 3D spheroid cultures of primary human hepatocytes in chemically defined conditions. Albrecht et al. (2019) predicted DILI in relation to oral doses and blood concentrations. Other studies relied on data collected in databases and used machine learning methods to derive predictive models. In particular, Hong et al. (2017) used a decision forest based on FDA's Liver Toxicity Knowledge Base for DILI prediction (ACC = 0.73, MCC = 0.33). Muller et al. (2015) used standard machine learning methods to predict DILI, relying on *in vivo* models of DILI of organic molecules.

The DILI prediction problem was previously investigated in two CAMDA challenges in 2018 and 2019. In CAMDA 2018, two human cell lines, MCF7 and PC3, were tested. Chierici et al. (2020) created a deep learning architecture for DILI prediction using these data. The authors obtained results slightly better than random ones—MCC equals 0.19 in the best case. Sumsion et al. (2020) solved the same problem using seven various classifiers. Prediction results were similar to the previous ones, with accuracy = 0.7 and MCC = 0.20. In 2019 CAMDA edition, three types of data, gene expression from human cell lines, chemical descriptors, and cell images, were provided by the organizers. The DILI definition was based on FDA DILI classification (Chen et al., 2016). We obtained AUC = 0.74 using SuperLearner methodology (van der Laan et al., 2007) in our study (Lesiński et al., 2021). Similar results were obtained in Liu et al. (2021).

The current study was performed within the framework of the CAMDA 2020 CMap Drug Safety Challenge. It aimed to develop predictive models for DILI, which would provide estimates of the risk of DILI for new substances using all available data sources: gene expression profiles in cancer cell lines exposed to them, their selected chemical properties, as well as their toxicological profiles.

## 2. Materials and Methods

### 2.1. Data

**Figure 1:**
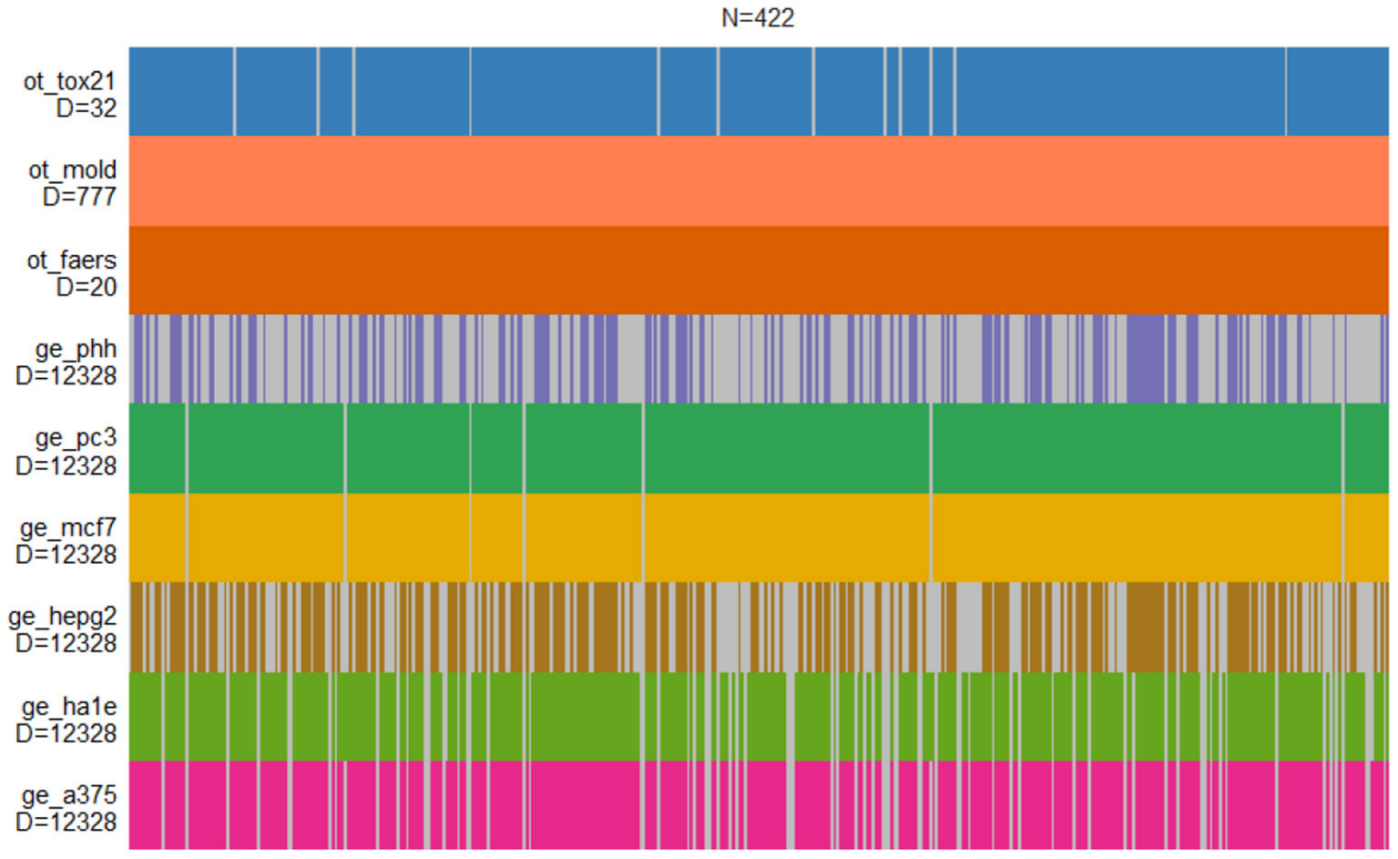
Structure of drug-induced liver injury (DILI) data sets. Each vertical bar corresponds to the compound that is present in a given set. Only MOLD and FAERS data sets contain information on all compounds.

The DILI classification is provided for nine independent data sets derived by CAMDA 2020 CMap Drug Safety Challenge. It contains six gene expression data sets from human cell lines, chemical descriptors of drugs, cell-based screening of pathway perturbations of the drugs, and information on reported DILI incidents from FDA FAERS database. The structure of the entire data set is shown in Figure 1.

The gene expression data for the study was generated using the L1000 Platform (Subramanian et al., 2017), developed for Connectivity Map (Lamb, 2007) at the Broad Institute. The Connectivity Map (also known as cmap) is a collection of genome-wide transcriptional expression data from cultured human cells treated with bioactive small molecules.

L1000 is a gene-expression profiling assay based on the direct measurement of a reduced representation of the transcriptome and computational inference of the portion of the transcriptome not explicitly measured. The abundance of ~1,000 landmark transcripts is measured directly. Eighty additional invariant transcripts are also explicitly measured to enable quality control, scaling, and normalization. Measurements of transcript abundance are made with a combination of a coupled ligase detection and polymerase chain reaction, optically addressed microspheres, and a flow-cytometric detection system. The expression of the remaining genes is inferred computationally from that of the measured ones.

The following human cell lines were used in the current study:

- A375: human melanoma—347 observations,
- HA1E: human embryonic kidney—347 observations,
- HPEG2: human liver cancer—235 observations,
- MCF7: breast cancer—415 observations,
- PC3: human prostate cancer—415 observations,
- PHH: primary human hepatocytes (currently considered to be the gold standard for hepatic *in vitro* culture models)—171 observations.

Chemical descriptors of drugs were computed with help of Mold2 program (Hong et al., 2008). Mold2 computes a large and diverse set of molecular descriptors encoding two-dimensional chemical structure information. Tox21 database (Huang et al., 2016) contains cell-based screening of pathway perturbations of the drugs. The FDA Adverse Event Reporting System (FAERS) (Kumar, 2019) is a database that contains information on adverse event and medication error reports submitted to FDA. The database is designed to support the FDA's post-marketing safety surveillance program for drug and therapeutic biologic products. Unfortunately, FAERS is not useful for predicting effects of new compounds.

Challenge organizers provided several alternative classifications of DILI based on two different classification schemes: DILI severity score and commercial status of the drug (Chen et al., 2016; Li et al., 2020). Additionally, two further DILI decisions were provided. They were later discovered to be controls for overfitting and for predictive potential of the approach used by participants. One was simply a random decision not connected to any descriptors whatsoever, another was a decision based on one of the molecular descriptors generated by Mordred. Altogether there were six different DILI scales provided to participants:

- DILI severity score (decision DILI2 in the challenge) (see Table 1);
- binary DILI severity score ≤ 6 (decision DILI1 in the challenge);
- Decision based on the commercial status of the drug (decision DILI4 in the challenge) with following classes: “withdrawn,” “box warning,” “warning and precaution,” “adverse event,” and “no match” (see Table 2);
- decision based on the commercial status of the drug (decision DILI3 in the challenge) with following binary classes: “withdrawn,” “box warning,” and “warning and precaution” vs. “adverse event” and “no match”);
- the artificial DILI class (decision DILI5 in the challenge) that was discovered to be a non-informative random decision (negative control);
- the artificial DILI class (decision DILI6 in the challenge) that was constructed using molecular weight of compound as decision (positive control).

**Table 1:** Number of objects in DILI2 classes.

**DILI severity**	**DILI category**	**Number**
0	no DILI	100
1	Steatosis	0
2	Cholestatsis; Steatohepatitis	26
3	Liver aminotransferases increase	138
4	Hyperbilirubinemia	24
5	Jaundice	38
6	Liver necrosis	3
7	Acute liver failure	26
8	Fatal hepatotoxicity	67

**Table 2:** Number of objects in DILI4 classes.

	**DILI category**	**Number**
1	Withdrawn	14
2	Box warning	18
3	Warnings and precautions	128
4	Adverse reactions	161
5	No match	101

### 2.2. Modeling

The modeling approach is based on the following general protocol:

- split the data into training and validation set;
- identify informative variables in the training set;
- select variables for model building;
- build model on training set;
- estimate model's quality both on training and validation set.

The procedure outlined above is cast within the 10 repeats of the 5-fold cross-validation scheme. In each repeat, the data set was split randomly into five parts in a stratified manner. Then, five models were developed for such a split using four parts as a training set and one part as a validation set. Each part served once as a validation set, and four times as part of the training set. This cross-validation was repeated 10 times with different random splits of data. The cross-validation allows to obtain an unbiased estimate of model quality using data unseen during feature selection and model building. Repeating the cross-validation is used to obtain an estimate of the distribution of results that are achievable with the procedure. Hence, it is instrumental in assessing what is the reasonable expectation of the performance on unseen data. We would like to stress that this estimation of the error of the model is a very important part of the procedure that is often overlooked in applications of machine learning.

The identification of informative variables was performed with the help of two methods: Welch *t*-test for differences in sample means (Welch, 1947), or multidimensional filter based on information theory developed in our laboratory (Mnich and Rudnicki, 2020) and implemented in the R package *MDFS* (Piliszek et al., 2019). MDFS allows to identify variables involved in non-linear and multidimensional interactions with the decision variable. Two variants of MDFS were used: one-dimensional (MDFS-1D) and two-dimensional (MDFS-2D). MDFS-1D is particularly apt for identifying variables that interact with decision variable in a non-linear fashion, whereas MDFS-2D facilitates the identification of the variables that gain importance due to interactions with other variables.

We used four popular classifiers for modeling: **Random Forest** algorithm (Breiman, 2001), **XGboost** (Chen et al., 2016), **Support Vector Machine (SVM)** (Cortes and Vapnik, 1995), and **logistic regression**. Random Forest and XGboost are based on decision trees and work well *out of the box* on most data sets (Fernández-Delgado et al., 2014). SVM is a machine-learning algorithm based on statistical learning theory. Logistic regression represents generalized linear models.

The prediction results based on individual data sets were combined into a single prediction, using the super learning methodology proposed by van der Laan et al. (2007). The super learner algorithm uses an internal cross-validation to obtain unbiased estimates of predictions from machine-learning models trained on particular data sets. First, Random Forest classifiers were built for each data set in the cross-validated manner to obtain the probability that a substance is harmful for the liver. Then, these probabilities were treated as new explanatory variables and used to build a second-order predictive model.

Five methods were applied to compute the combined model:

1. linear combination with non-negative weights, computed with the NNLS algorithm (Lawson and Hanson, 1995);
2. choice of the best-performing single classifier;
3. mean of all the results;
4. mean of five best results;
5. applying Random Forest machine-learning algorithm.

One may note that methods (2–4) are special cases of non-negative linear combination, although optimized in different way than method 1. In particular, a simple mean of all the elementary results usually leads to the improvement of the predictions, when all the classifiers perform equally well. Contrastingly, the choice of the best single model or the average of a few top-rated ones is the optimal method when some classifiers definitely outperform the others. The NNLS method takes into account dependencies between the base results, while Random Forest gives the opportunity to explore nonlinear and multivariate interactions. However, in the absence of strong interactions, both more sophisticated methods may not outperform the simple ones.

To increase the stability of the combined results, we performed 10 repeats of the 5-fold internal cross-validation and built 10 separate combined models for each method. The final results are the averages of the predictions over the cross-validation loops. The entire super learning protocol (including feature selection, building of the elementary machine learning models, computing the component predictions, and combining them into the final results) was tested in 10 repeats of 10-fold external cross-validation.

### 2.3. Modeling Details

In the first stage, models were created using each data set separately for the four binary decisions provided by the organizers. For each data set, we used all possible combinations of three feature selection filters and classification algorithms—nine models for each binary decision on each data set. MDFS was used with default parameters. Unfortunately, feature selectors did not find relevant variables in cell-line data sets. In the presence of strongly correlated variables, with only weak association with the decision variable, the truly relevant variables may not be discovered due to corrections for multiple test applied in computation of statistical significance. The correction is based on the formal number of degrees of freedom, i.e., number of variables, whereas the true number of independent variables may be orders of magnitude lower. Therefore, models were built using 100 most informative variables for large data sets (human cell lines and mold) and all variables for FAERS and tox21.

## 3. Results and Discussion

All analysis were performed in 10 repeats of 5-fold cross-validation scheme. Tests on human cell lines gene expression were carried in two ways: models were built using either all or only explicitly measured base variables. Initial feature selection were performed by MDFS in one and two dimensions way and Welch *t*-test. Best prediction results were obtained by models based on MDFS 1D. The final models were built using MDFS 1D.

### 3.1. Models for Individual Data Sets on Four Predefined Decision Variables

The results of this stage are collected in Table 3. The results obtained for two decision variables (DILI5 and DILI6) serve as a gauge of robustness of the modeling procedure—DILI5 examines whether a modeling procedure results in overfitted models and DILI6 examines whether it can find a strong signal present in the data. Two other decision variables (DILI2 and DILI4) represent two alternative definitions of DILI, one that is based on the severity of biological effects and the other that is based on market status.

**Table 3:** Results (AUC) of prediction for binary drug-induced liver injury (DILI) endpoints.

**Data set**	**DILI1**	**DILI3**	**DILI5**	**DILI6**
MOLD	0.56 ± 0.06	0.69 ± 0.04	0.52 ± 0.05	0.99 ± 0.01
FAERS	0.64 ± 0.07	0.63 ± 0.06	0.50 ± 0.04	0.62 ± 0.06
tox21	0.51 ± 0.07	0.61 ± 0.06	0.47 ± 0.06	0.76 ± 0.06
	**GE all**			
A373	0.56 ± 0.08	0.49 ± 0.07	0.52 ± 0.06	0.57 ± 0.07
HA1E	0.53 ± 0.09	0.53 ± 0.06	0.48 ± 0.06	0.56 ± 0.06
HEPG2	0.57 ± 0.09	0.55 ± 0.08	0.52 ± 0.08	0.64 ± 0.08
MCF7	0.50 ± 0.06	0.54 ± 0.06	0.47 ± 0.05	0.62 ± 0.05
PC3	0.49 ± 0.06	0.55 ± 0.05	0.45 ± 0.05	0.58 ± 0.05
PHH	0.48 ± 0.13	0.49 ± 0.09	0.49 ± 0.09	0.54 ± 0.09
	**GE base**			
A375	0.56 ± 0.08	0.50 ± 0.07	0.51 ± 0.06	0.55 ± 0.07
HA1E	0.52 ± 0.09	0.52 ± 0.07	0.49 ± 0.05	0.57 ± 0.07
HEPG2	0.58 ± 0.09	0.52 ± 0.07	0.51 ± 0.08	0.63 ± 0.08
MCF7	0.48 ± 0.06	0.54 ± 0.06	0.49 ± 0.06	0.61 ± 0.07
PC3	0.55 ± 0.07	0.54 ± 0.05	0.48 ± 0.05	0.57 ± 0.05
PHH	0.49 ± 0.12	0.51 ± 0.08	0.49 ± 0.10	0.53 ± 0.10

#### 3.1.1. Controls for the Robustness of the Modeling Procedure

The results for the artificial endpoint DILI5 clearly show that our learning procedure is robust and does not lead to overfitting: the models for a random variable lead to random results. The more interesting results were obtained for the DILI6 endpoint. First, nearly all data sets carried some information about this endpoint, with two exceptions—the PHH cells were non-informative and the MOLD data set produced a perfect model. Initially, we suspected some data corruption in the MOLD data set. After informing the organizers, we have learned that the DILI6 endpoint was constructed from molecular mass of the compound, and was meant to be a positive control for the modeling procedure. Interestingly, despite artificial construction of this decision, a weak predictive signal could be found in all data sets, showing that the molecular mass of the compound correlates with toxicity (tox21 data set), frequency of DILI incidence (FAERS data set), and influence on gene expression. The correlation of effect in cell-lines with mass can be explained by a simple observation—the large molecules are more likely to be metabolized to molecular fragments that trigger some response in the cell. It is very interesting that the only cell line derived from healthy hepatocytes is not affected by the molecular mass of compound. It likely happens that healthy hepatocytes are specialized in dealing with nasty chemicals and do not need to change the metabolism to cope with them—they are constantly ready.

#### 3.1.2. Results for Two Alternative Binary Definitions of DILI

**Figure 2:**
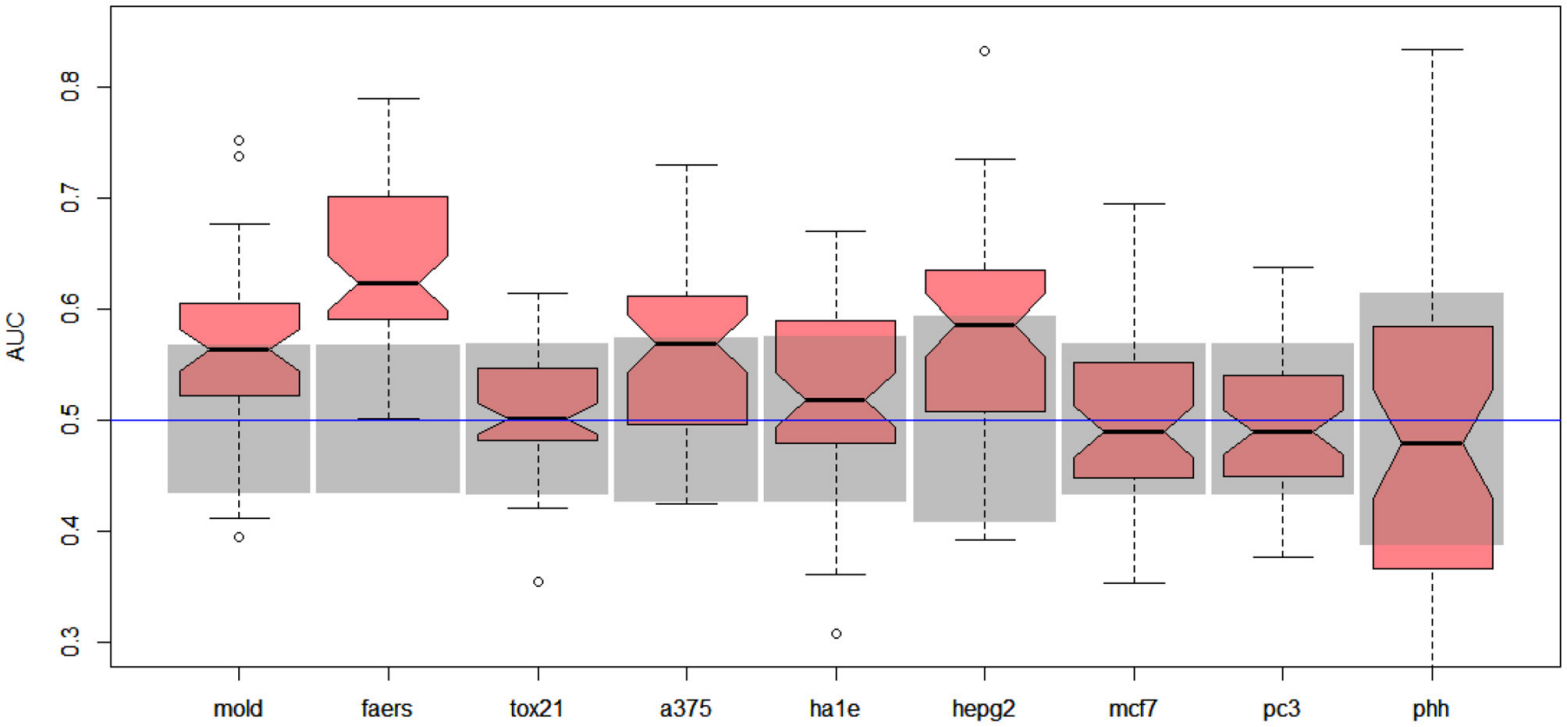
Results for DILI1 decision (DILI severity 6 or above) on various data sets. Gray boxes correspond to 95% confidence interval for the null hypothesis. Predictions based on MOLD, FAERS, A375, and HEPG2 are better than random ones.

Unfortunately, the only predictive model for DILI1 (AUC = 0.64) was built on FAERS data. This result is useless for the prediction of DILI, since the FAERS data set is constructed from statistics of DILI reports for a given compound. Among the remaining models only the ones built using MOLD, as well as A375 and HEPG2 human cell lines achieved non-random (albeit very weak) predictions (see gray boxes in Figure 2. Models built using other data sets were within bounds of statistical error.

**Figure 3:**
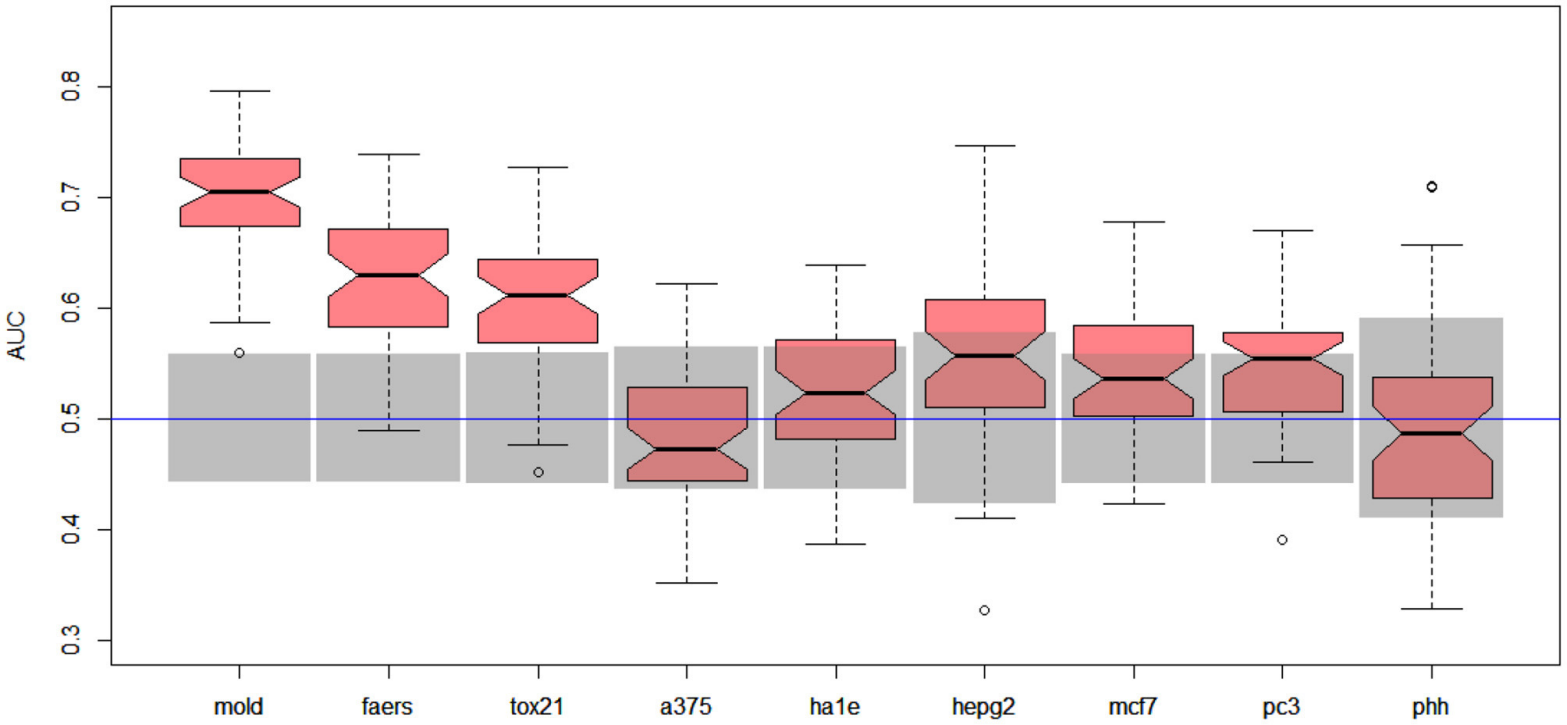
Results for DILI3 decision (“withdrawn,” “box warning,” and “warning and precaution” vs. “adverse event” and “no match”) on various data sets. Gray boxes correspond to 95% confidence interval for the null hypothesis. Predictions based on MOLD, FAERS, and tox21 are significantly better than random ones. Results for HEPG2, MCF7, and PC3 are on border between random and non-random ones.

Better results were obtained for DILI3 decision (see Figure 3). MOLD was the most informative data set (AUC = 0.69). Non-random models were also obtained using FAERS and tox21 data sets. Unfortunately, all models obtained with the help of gene expression profiles were non-informative.

#### 3.1.3. Comparison of Four Machine Learning Algorithms

The entire modeling workflow for all decision variables was executed with four different algorithms used for generating predictive models: Random Forest, XGBoost, SVM, and logistic regression. For each data set, and each decision variable, the highest AUC was obtained for models built with Random Forest. Slightly worse results were obtained with XGBoost and SVM and the worst ones with logistic regression. Representative results are displayed in Table 4.

**Table 4:** Different classifiers comparison (AUC) on chosen data sets for DILI1 and DILI3.

**Data set**	**Random forest**	**XGBoost**	**Logistic regression**	**SVM**
	**DILI1**			
MOLD	0.56 ± 0.06	0.54 ± 0.06	0.52 ± 0.07	0.55 ± 0.05
A375	0.56 ± 0.08	0.54 ± 0.07	0.53 ± 0.08	0.54 ± 0.05
HEPG2	0.57 ± 0.09	0.55 ± 0.09	0.54 ± 0.09	0.55 ± 0.08
MCF7	0.50 ± 0.06	0.50 ± 0.07	0.51 ± 0.07	0.49 ± 0.07
	**DILI3**			
mold	0.69 ± 0.04	0.67 ± 0.05	0.65 ± 0.04	0.66 ± 0.04
tox21	0.61 ± 0.06	0.61 ± 0.06	0.59 ± 0.05	0.60 ± 0.05
HEPG2	0.55 ± 0.07	0.54 ± 0.07	0.53 ± 0.08	0.55 ± 0.08
MCF7	0.54 ± 0.06	0.52 ± 0.06	0.52 ± 0.07	0.51 ± 0.07

### 3.2. Decision Scanning

**Figure 4:**
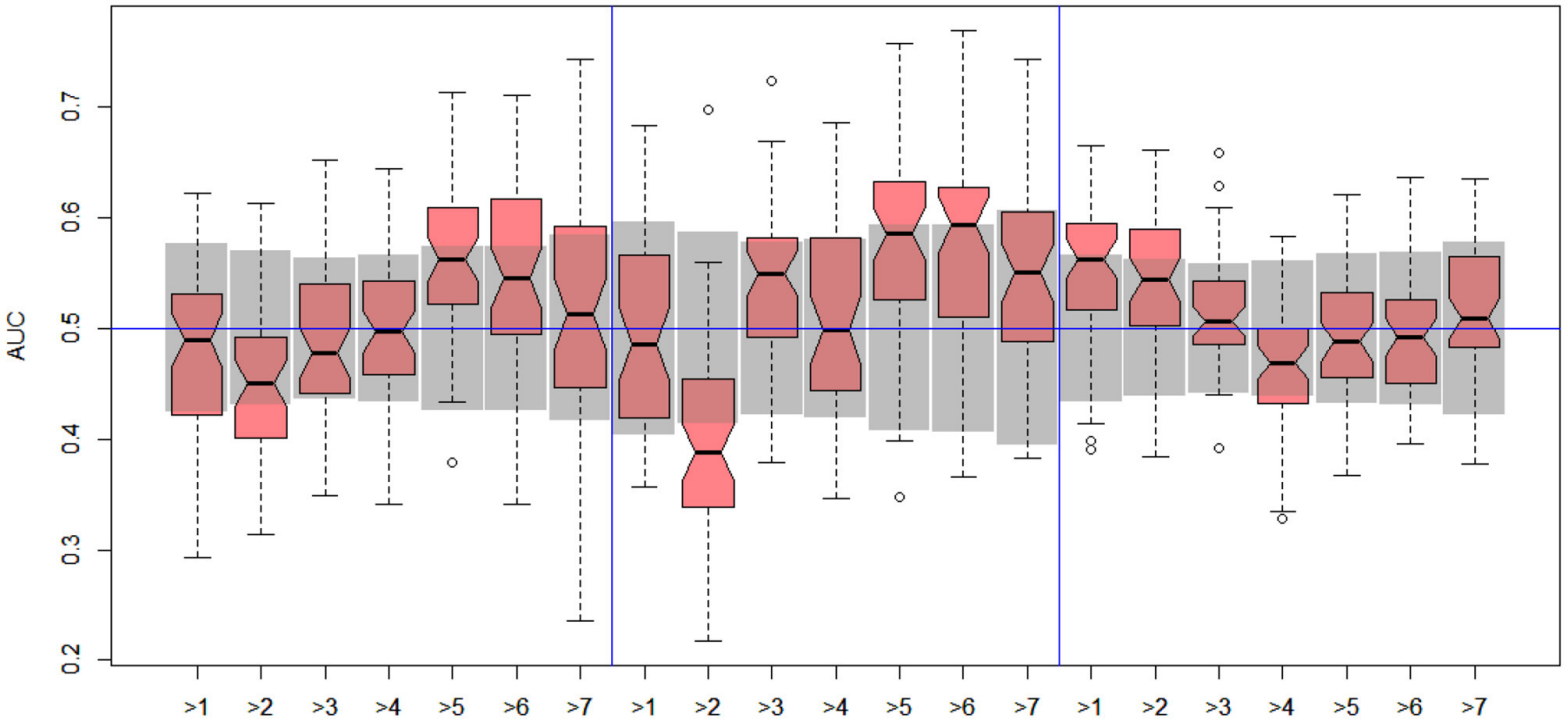
DILI2 scanning results for A375 **(left)**, HEPG2 **(middle)**, and MCF7 **(right)** cell lines. Gray boxes correspond to 95% confidence interval for the null hypothesis.

**Figure 5:**
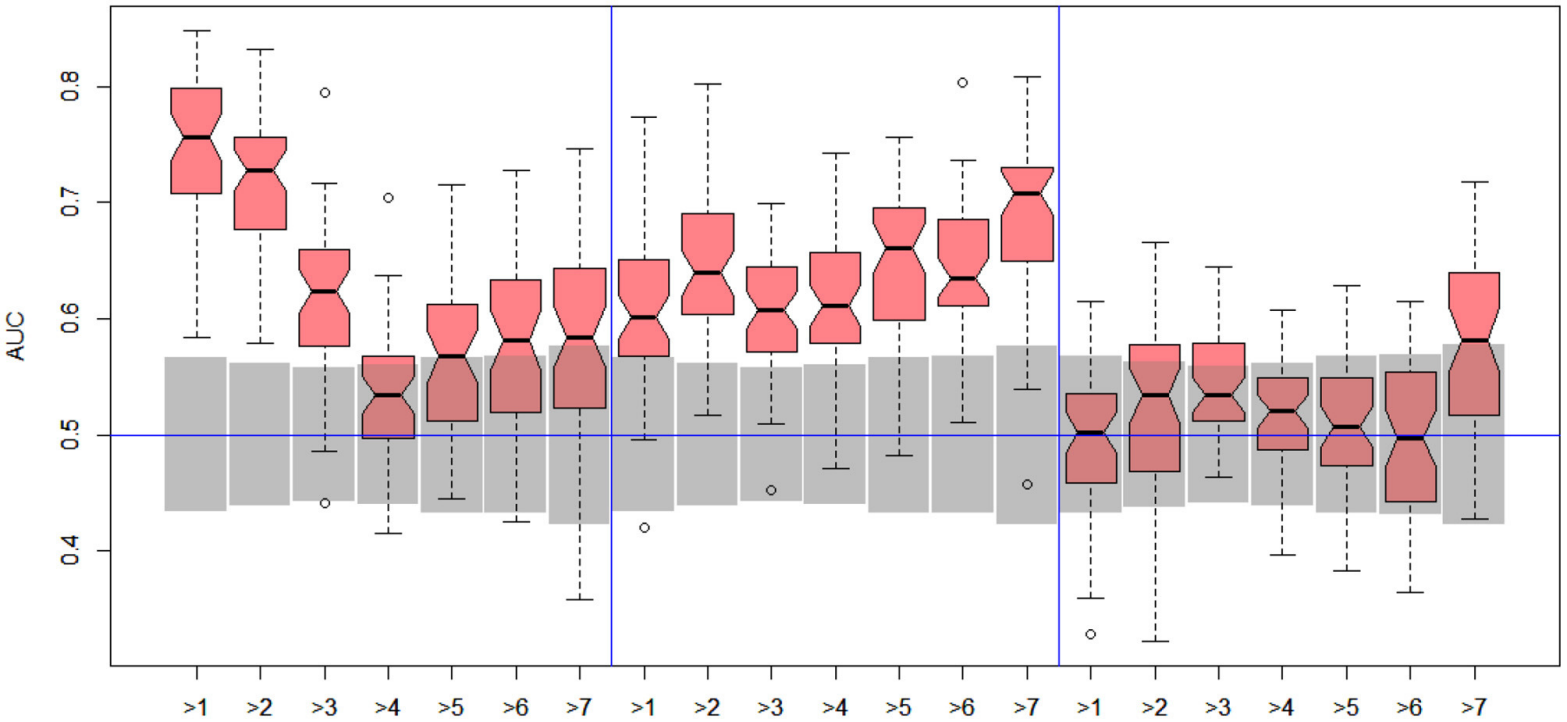
DILI2 scanning results for MOLD **(left)**, FAERS **(middle)**, and tox21 **(right)** data sets. Gray boxes correspond to 95% confidence interval for the null hypothesis.

**Figure 6:**
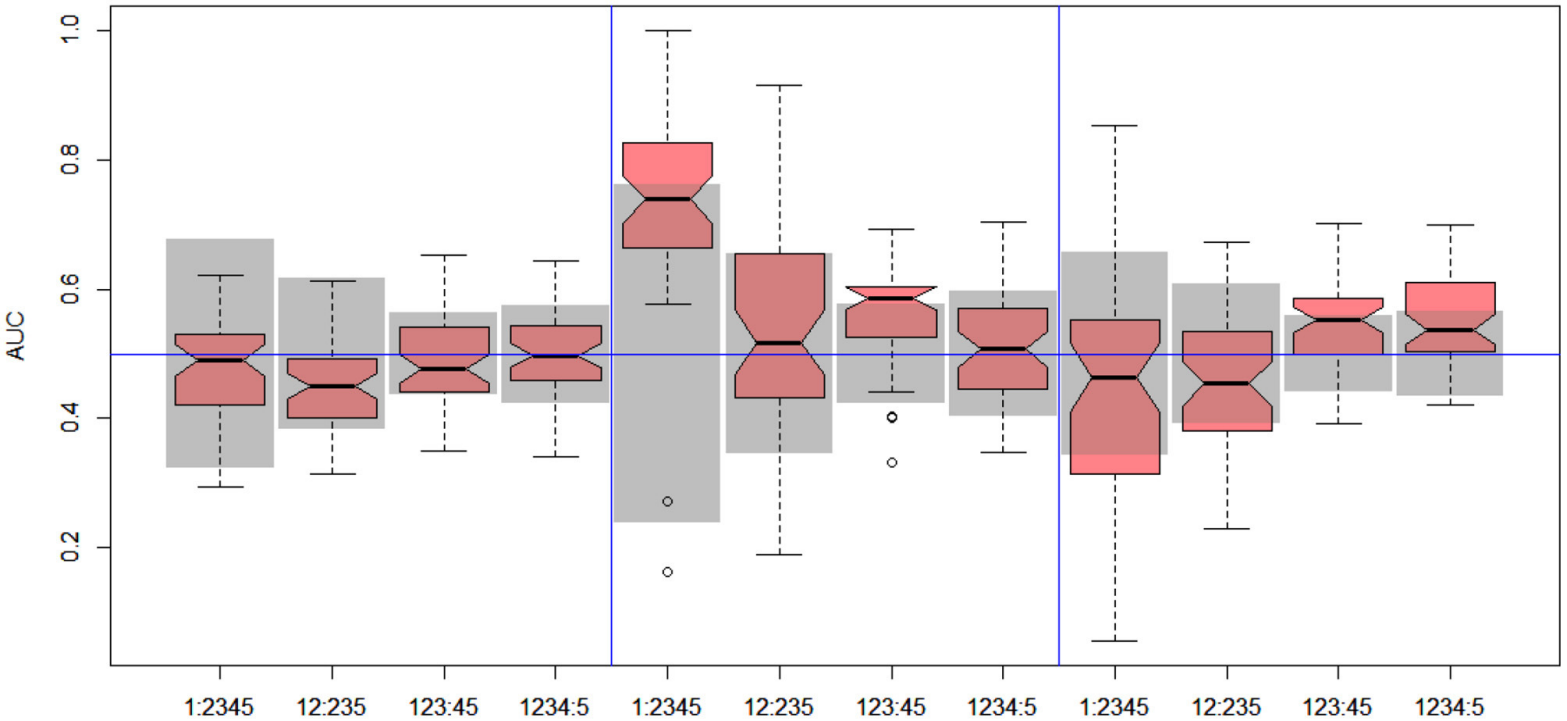
DILI4 scanning results for A375, HEPG2, and MCF7 cell lines. Gray boxes correspond to 95% confidence interval for the null hypothesis.

**Figure 7:**
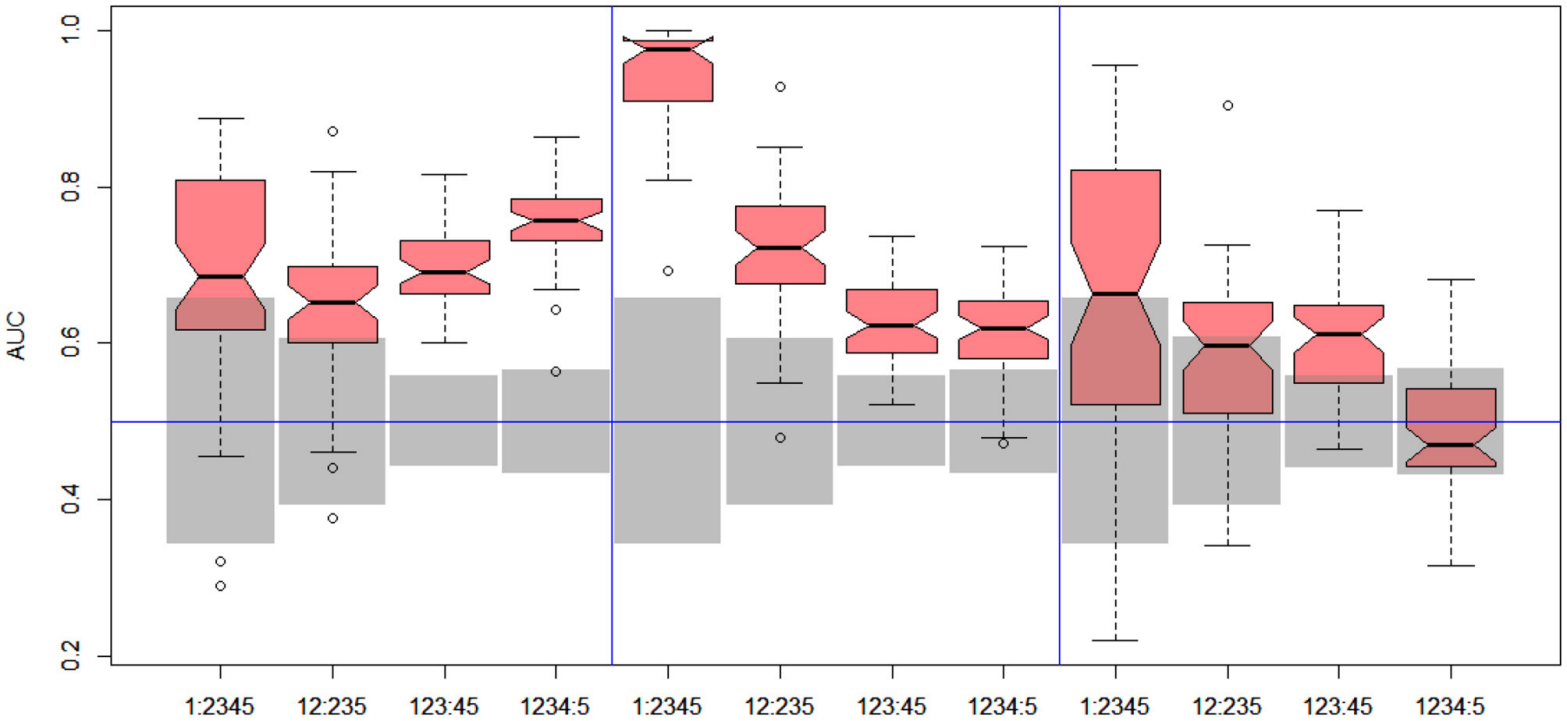
DILI4 scanning results for MOLD, FAERS, and tox21. Gray boxes correspond to 95% confidence interval for the null hypothesis.

In the next stage, we explored all possible binary divisions for DILI severity (DILI2) and DILI decision based on market status (DILI4). All results for DILI severity (decision DILI2) are displayed in Table 5, and for the classification based on the market status of the drug (decision DILI4) in Table 6. Figures 4, 5 show cross-validation results for the DILI2 decision for the selected human cell lines and other data sets, respectively. Figures 6, 7 show corresponding results for DILI4 decision.

**Table 5:** AUC for different binary decisions based on drug-induced liver injury (DILI) severity.

**Data set**	**>1**	**>2**	**>3**	**>4**	**>5**	**>6**	**>7**
MOLD	**0.75** **±** **0.05**	0.72	0.62	0.53	0.56	0.57	0.57
FAERS	0.61	0.64	0.61	0.61	0.64	0.64	0.69
tox21	0.49	0.52	0.54	0.51	0.50	0.50	0.57
A375	0.47	0.45	0.49	0.49	0.56	0.55	0.51
HA1E	0.52	0.47	0.50	0.53	0.52	0.50	0.57
HEPG2	0.49	0.48	0.55	0.52	0.57	0.58	0.56
MCF7	0.56	0.55	0.52	0.47	0.49	0.49	0.52
PC3	0.50	0.52	0.50	0.50	0.49	0.47	0.49
PHH	0.50	0.53	0.48	0.44	0.49	0.49	0.45

*Most significant result (with error) is in bold*.

**Table 6:** AUC for different binary decisions based on market status of the compound.

**Data set**	**1 : 2345**	**12 : 345**	**123 : 45**	**1234 : 5**
MOLD	0.69	0.64	0.70	**0.75** **±** **0.05**
FAERS	0.95	0.72	0.62	0.61
tox21	0.66	0.58	0.60	0.50
A375	0.45	0.52	0.52	0.52
HA1E	0.52	0.52	0.51	0.46
HEPG2	0.53	0.55	0.59	0.52
MCF7	0.53	0.53	0.54	0.53
PC3	0.55	0.44	0.55	0.49
PHH	0.51	0.55	0.54	0.46

*Labels: 1: “withdrawn,” 2: “box warning,” 3: “warning and precaution,” 4: “adverse event,” 5: “no match”. Most significant result (with error) is in bold*.

#### 3.2.1. DILI Severity Scale

In the case of DILI2 decision, the best result (AUC = 0.75, MCC = 0.36, ACC = 0.80) was obtained for MOLD data and division “no DILI” vs. all DILI severity degrees. Prediction quality based on chemical description decreased with the inclusion of more harmful substances to the “no DILI” class. Relatively good and stable results were obtained for models built using the FAERS data set. They reach maximum for the model that discerned compounds from the highest severity class from all others. However, as mentioned earlier, models based on FAERS are useless for new compounds. Tox21-based models were not statistically better than a random model for all possible binary divisions with the exception of the split separating highest level of DILI severity from other classes. Nonetheless, even in this case the model is very weak.

The models built on human cell lines gene expression and toxicology scores were mostly non-informative. Weak predictive models were obtained for HEPG2 lines for two binary divisions, and for and MCF7 for discerning between harmless compounds against all other classes. One should stress that the quality of these models is dependent on the border between weakly predictive and random ones.

#### 3.2.2. DILI Market Status

In the case of DILI4 decision, models built on FAERS data almost perfectly predicted decision “withdrawn” vs. “all others.” They also worked relatively well for discerning the two highest levels from all others. This is not surprising, since the decisions on withdrawal of the drug from the market are based on the FAERS database. Models obtained using chemical descriptors also achieved a relatively good quality with AUC, varying between 0.64 and 0.75. Unfortunately, almost all models using gene expression profiles of human cell lines are non-informative. Only the model using HEPG2 in two middle splits of DILI decision achieved AUC significantly better than a random one.

### 3.3. Composite Models

In the final stage of the study, the results of the predictive models based on single data set were combined into a single model by means of the super learning approach. This methodology significantly improved our results in a previous DILI prediction study (CAMDA2019; Lesiński et al., 2021). This procedure includes verification of the results by cross-validation, hence entire modeling procedure described earlier had to be repeated multiple times within a cross-validation loop. The super learning models were built using those splits of both DILI2 and DILI4 decisions for which more than one informative model had been obtained. As mentioned earlier, the FAERS data set is useless for formulating predictions for new compounds. Hence it was not used in super learning. Unfortunately, super learning did not improve the results for current data sets—the AUC of prediction from Super Learner was not significantly better than the results obtained on the best single data set.

There are several possible explanations for this rather disappointing result. The models based on gene expression are generally rather weak, in most cases significantly weaker than models based on the molecular descriptors. In the previous edition of CAMDA toxicology challenge, the molecular descriptors were merged with gene expression profiles to create unified models that were then merged with the super learning. In the current edition, adding expression profiles to molecular descriptors did not improve results compared to molecular descriptors alone. What is more, the models have a relatively good predictive power only for a single line, namely the HEPG2. Finally, while the molecular descriptors were available for all compounds, the number of gene expression profiles for compounds varied between 171 for PHH, 235 for HEPG2, and up to 415 for MCF7. The quality of ML models falls with decreasing number of objects, hence any possible gains due to adding a weak signal from gene expression model to the stronger signal from the molecular model were offset with falling strength of molecular model in a smaller data set.

### 3.4. Summary and Discussion

The main findings of the study can be summarized as follows:

- The modeling procedure applied in the current study is robust and can build predictive models where the information about the decision variable is present in the descriptors.
- The toxicology data set is not useful for building predictive models.
- The FAERS data set reflects the market status of drugs to some extent. A very good predictive model can be obtained for the *Withdrawn* class. On the other hand, models for the remaining classes are weak, showing that rules guiding assignment to different classes are not based on FAERS data alone and take into account other factors.
- The methods for pre-processing the data available in the gene expression of perturbed cell lines can have profound effect on the quality of the models based on these data. No informative models were obtained with the data used in the current edition of the challenge, while useful models could be obtained on the smaller data sets available in the previous challenge.
- Scanning of two DILI severity scales has shown that the best models can be obtained for discerning between “no-DILI” and “DILI” classes. The models are not precise enough to discern different levels of DILI severity.

The last finding is the most important result of the study and therefore merits more detailed discussion. The best predictive models in the current study were obtained for classifiers discerning drugs classified as “non-DILI,” from all others in both DILI classification scales. The sets of “non-DILI” compounds classified according the DILI severity scale and according to market status are nearly identical (they differ by a single compound that has a *non match* market status and DILI severity index 3). Consequently, the predictions for these two sets are also nearly identical, with AUC = 0.75. Despite this relatively low predictive power of the model, its predictions could be used to guide early phases of drug development by allowing to concentrate development on the compounds with a lower risk of DILI. For example, one may split the set of compounds in two equal classes *lower DILI risk* and *higher DILI risk*. In such a case, the former set would consist of 36% non-DILI and 64% DILI compounds, while the latter would consist of 11% non-DILI and 89% DILI compounds. That is more than 3-fold enrichment of non-DILI compounds in the *lower DILI risk* class.

The results obtained in the current challenge have comparable quality to those obtained in the 2019 CAMDA challenge with identical methodology. However, there are two main differences in comparison with the previous challenge. First, the model based on the molecular data alone is significantly better than the model obtained in the previous challenge (AUC = 0.75 vs. AUC = 0.66). This can be attributed to a much larger data set in the current challenge (422 vs. 233 compounds) and in particular in the “no-DILI” class (100 vs. 54 compounds).

On the other hand, the results were significantly worse for the cell lines in general, and the MCF7 cell line in particular. In the previous challenge, the AUC for classifier built using the MCF7 data set alone was 0.62 and was statistically significant. In the current challenge, the AUC for the analogous classifier was 0.56 and it was statistically not significant. That decrease was observed despite a markedly larger data set. The probable reason behind the difference in the quality of the results may be due to the differences in data pre-processing. In many cases, the LINCS database contains multiple samples for the single compound. In the previous challenge, we explored the quality of the models developed when different methods of pre-processing were applied. We determined that the best results were obtained when a single sample selected randomly from those incubated for 24 h was used. Attempts to remove noise in the data by using a signal from multiple probes resulted in the significant decrease of performance. We suspect that samples presented in the current challenge were obtained by some noise removing procedure that unfortunately lead to the removal of the signal. The weak performance of the models based on the cell-line data was a reason why both methods of combining the gene expression data with molecular descriptors did not improve the results.

## 4. Conclusions

The DILI classification is a complex process that takes into account multiple factors. The most important are as follows: the severity of harmful effects, the severity of the medical condition for which the drug is applied, and the length of treatment. What is more, the drug that has small effects in a short time-scale, when used for years it may have a much stronger cumulative DILI effects than a drug that is taken only sporadically. These factors significantly constrain the predictive ability of the models. Nevertheless, relatively good predictive models for DILI can be obtained using molecular properties of these compounds. While the resulting models are not good enough for making meaningful predictions for individual compounds, they can be used to split compounds into two classes, “lower DILI risk” and “higher DILI risk,” with more than 3-fold enrichment of “non-DILI” compounds in the former in comparison with the latter.

## Data Availability Statement

The CMap Drug Safety Challenge data can be downloaded from the CAMDA 2020 Website: http://camda2020.bioinf.jku.at/ (accessed in April 2020).

## Author Contributions

WL: methodology, software, investigation, visualization, and writing—original draft. KM: methodology, formal analysis, software, investigation, visualization, writing—original draft, and writing—review and editing. WR: conceptualization, funding acquisition, methodology, writing—original draft, and writing—review and editing. All authors read and approved the final manuscript.

## Conflict of Interest

The authors declare that the research was conducted in the absence of any commercial or financial relationships that could be construed as a potential conflict of interest.
